# Paradigm-shift: radiological changes in the asymptomatic iNPH-patient to be: an observational study

**DOI:** 10.1186/s12987-018-0090-9

**Published:** 2018-02-09

**Authors:** D. C. Engel, S. D. Adib, M. U. Schuhmann, C. Brendle

**Affiliations:** 1 Department of Neurosurgery, University Hospital of Tuebingen, Hoppe-Seyler-Strasse 3, 72076 Tuebingen, Germany; 2 Department of Neuroradiology, University Hospital of Tuebingen, Hoppe-Seyler-Strasse 3, 72076 Tuebingen, Germany

**Keywords:** Etiology, Pathophysiology, Idiopathic normal pressure hydrocephalus, Tight high convexity, Ventriculomegaly, Evan’s index

## Abstract

**Background:**

Many radiological signs are known for the diagnosis of idiopathic normal pressure hydrocephalus (iNPH). However, there is little information about these signs in the pre-symptomatic phase. For pathophysiological investigative purposes we conducted a descriptive image analysis study on pre-symptomatic patients.

**Methods:**

Patients that had contact with either the neurological or neurosurgical department of the university hospital Tuebingen from 2010 through 2016 with magnetic resonance images > 3 years before onset of symptoms, were included. The date of onset and severity of symptoms, date of first imaging and birth date were recorded. Evan’s index (EI), width of the third ventricle (3VW), tight high convexity (THC), Sylvian fissure, extent of white matter hyperintensities and aqueductal flow were assessed in images before and around symptom onset.

**Results:**

Ten patients were included. In all ten patients the first symptom was gait disturbance. Nine of ten pre-symptomatic images showed classic signs for iNPH. EI showed a significant increase between the pre-symptomatic and symptomatic phase. 3VW showed a trend for increase without significance. THC changed back and forth over time within some patients.

**Conclusions:**

In accordance with the scarce literature available, radiological changes are present at least 3 years before onset of iNPH-symptoms. EI seems to be a robust measure for pre-symptomatic radiological changes. Extrapolating the data, the development of iNPH typical changes might be an insidious process and the development of THC might be a variable and non-linear process. Further studies with larger sample sizes are necessary to put these findings into the pathophysiological perspective for the development of iNPH.

## Background

The etiology of idiopathic normal pressure hydrocephalus (iNPH) has been a research focus since its first description in 1965 [1]. The overall prevalence is estimated to be 10.2–31.4/100,000, and might even be 5900/100,000 in the > 80 year olds [2–4]. Better understanding of the pathophysiology and better treatment possibilities are needed for our aging populations.

The pathophysiology of iNPH is not completely understood currently. Studies indicate that a prolonged, possibly lifelong process underlies the development of what we know as iNPH [5–7]. Known changes in pathophysiological parameters measured at the time of symptomatic iNPH include decreased subcortical cerebral blood flow (CBF) [8], decreased flow in the internal carotid artery [9], reduced flow velocity in the superior sagittal sinus [10], a higher incidence of retrograde jugular flow during the Valsalva maneuver [11], increased aqueductal cerebrospinal fluid (CSF) flow velocity [12–14], decreased cervical CSF stroke volume [9], decreased elastance of the intradural compartment and increased CSF pulsatility and outflow resistance [15, 16], increased lactate concentrations in the lateral ventricles [17], deep and (partially reversible) periventricular white matter hyperintensities (WMH) [18–20] and increased neurofilament light chain protein in CSF as a sign of neurodegeneration [21, 22].

The hydraulic press hypothesis of Hakim and Adams explains the development of iNPH by disturbance of CSF-resorption leading to an increased outflow resistance, which in turn leads to an initial increase of ICP. The authors postulate that due to the increased ICP the ventriculomegaly occurs from the indirect linearity of pressure and surface at constant pressure according to Pascal’s Law [23]. However, it lacks an explanation for the asymptomatic phase of increased ICP. Furthermore, a transmantle pulsatile gradient within the CSF of the cerebral ventricles in symptomatic iNPH patients could not be proven [24].

Another theory exists called the two-hit hypothesis, comprised of the occurrence of a benign external hydrocephalus during infancy followed by ischaemic insults leading to deep and periventricular WMH [7]. The first hit results in a larger intracranial volume in iNPH patients [7]. The second hit, ischemic insults, could be induced by worsening of periventricular ischemia [25, 26], softening of the brain [27], or decreased CSF resorption [28, 29] leading to the beginning of symptoms [7].

A third, hydrodynamic, hypothesis of Greitz is explained as “restricted arterial pulsation hydrocephalus” or “increased capillary pulsation hydrocephalus” [30]. An increasing rigidity of the cerebral arteries by arteriosclerosis could lead to the inability to attenuate the pulsations of CBF into the brain parenchyma, causing a reduction of the periventricular metabolism and degeneration of the periventricular parenchyma [30–32].

In addition to the above-mentioned mechanical hypotheses, genetic predispositions such as homozygous apolipoprotein E allele on chromosome 19, play an important role in familial iNPH [33–35].

The above-mentioned pathophysiological parameters cannot prove any of the stated hypotheses, because all data have been logically derived in patients who already had symptomatic iNPH. Therefore we aimed to investigate the presence of iNPH-typical changes in diagnostic images in the pre-symptomatic phase of iNPH.

## Methods

### Patient inclusion

The diagnosis of iNPH was screened as either probable or possible and secured according to international guidelines by an objective improvement of gait in the 10 m walking test after either (repetitive) CSF tap-test(s) or lumbar drainage for 3 days [36–39]. Those patients with imaging available 3 or more years before symptom-onset, that had contact with the neurological or neurosurgical department of the university hospital Tuebingen for the diagnosis of iNPH from 2010 thru 2016 were included. Time of symptom-onset was extracted from the medical history. The 3-year cut-off was chosen because of the insidious onset of iNPH. Patients with an unclear hydrocephalus-type, possibly due to neurotrauma, meningitis or intracranial bleeding or with an unclear symptom-onset or origin, i.e. due to simultaneous high-grade cervical spinal stenosis were excluded. Age, time of symptom onset, time of first imaging and first presenting symptoms were assessed. All of the imaging data (MRI and CT) were included in this retrospective analysis.

### Image analysis

Imaging data were acquired on CT and MRI scanners approved for clinical routine use. Data were reconstructed according to clinical standard orientated at the corpus callosum in the axial slices and at the brain stem in the coronal slices. For image analysis in CT, the axial slices and where available the coronal slices were assessed. For image analysis in MRI, the axial fluid attenuated inversion recovery (FLAIR) sequence and where available the axial, coronal or sagittal T2-weighted images were assessed. Images after treatment by ventriculoperitoneal shunt implantation (VPS) were not included.

A neuroradiologist (CB) scored the imaging data being blinded for the hypotheses of this project and the clinical data. The following measurements were obtained:

1. Evan’s index (EI) was determined as the ratio of the maximum transverse diameter of the frontal horns of the lateral ventricles and the diameter of the inner skull [40].
2. The width of the third ventricle (3VW) was measured in the axial plane in the mid-portion of the third ventricle.
3. Tight high convexity (THC) of the subarachnoid space was measured following the method of Narita et al. [41] in both transverse and coronal sections and rated as: 0, normal; 1, mild tightness; 2, moderate tightness; 3, severe tightness.
4. Width of the Sylvian fissure was assessed only on transverse sections as: 0, narrowed, 1, normal; 2, mildly dilated; 3, severely dilated [41].
5. Periventricular and deep WMH were scored according to Fazekas [42].
6. The existence of aqueductal flow voids/jets was documented if appropriate MR-sequences were available.

The callosal angle was not included due to missing coronal sections in many of examinations.

### Statistics

Normal distribution of the data was denied by the Shapiro–Wilk test. Wilcoxon text was performed to compare EI and 3VW before and after onset of symptoms in patients with available imaging data at both time points.

## Results

From 2010 through 2016, 382 patients suspected of having NPH had contact with either the neurological or neurosurgical department. Ten patients met inclusion criteria and were finally included in the study with a male-to-female ratio of 1:1 and a mean age at symptom-onset of 77.4 years (range 70.9–88.7).

### Clinical data

Seven patients were treated by VPS and improved long-term (> 1 year); the other 3 either refused treatment or received repetitive lumbar punctures. Gait disturbance was the first reported symptom in all cases. Nine patients suffered all three symptoms of the Hakim triad at the time of diagnosis. Only one patient reported no cognitive deficits and had a Mini Mental State Examination (MMSE) score at time of diagnosis 30/30. The other patients did indicate cognitive deficits. In this group MMSE ranged from 12 to 29/30. Nine patients were diagnosed with hypertension before symptom-onset.

### Imaging data

**Fig. 1:**
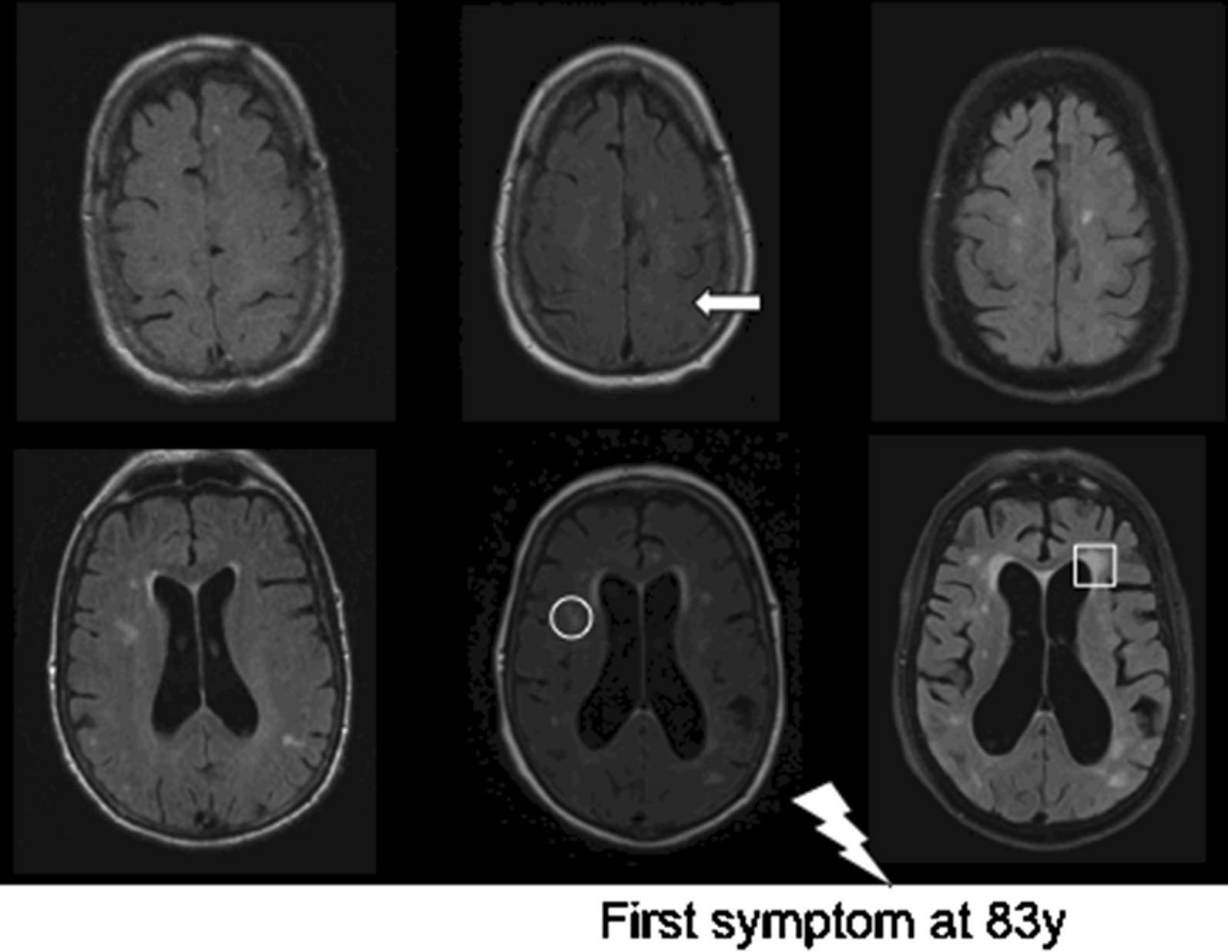
Pre- and post-symptomatic magnetic resonance images of a patient that developed symptoms after 10 years. The MRI images show the changes in ventricle width, deterioration of white matter hyperintensities (circle: deep; square: periventricular) and tight high convexity (arrow) all before onset of symptoms (thunderbolt) of a typical case. Left: 10 years before symptom onset. Middle: 4 years before symptom onset. Right: 6 months after symptom onset

On average the first imaging examination (CT or MRI) was conducted − 6.2 years to symptom onset (range 3.1–10.3). In 2 cases 3 MRIs were available at − 6.1, − 2.8 and 0.2 and − 10.3, − 4.1 and 0.5 years to symptom onset respectively. All time points are relative to symptom onset. Details of the available imaging data are shown in Table 1. An illustrative case (patient 7) with increasing radiological changes from the pre-symptomatic to symptomatic phase is shown in Fig. 1.

**Table 1:** Images available per patient

Patnr	Imaging 1		Imaging 2		Imaging 3		Imaging 4	
	Timepoint	Modality	Timepoint	Modality	Timepoint	Modality	Timepoint	Modality
1	− 4.7	CT	− 3.3	CT	− 0.9	MRI	2.1	CT
2	− 5.4	CT	− 0.1	CT	0.2	MRI		
3	− 6.1	MRI	− 2.8	MRI	0.2	CT	1.9	CT
4	− 3.1	CT	0.9	CT				
5	− 3.6	CT	− 0.1	MRI				
6	− 7.8	MRI	1.5	MRI	1.9	CT		
7	− 10.3	MRI	− 4.1	MRI	− 1.4	CT	0.5	MRI
8	− 6.1	MRI	0.7	MRI	2.0	CT		
9	− 9.2	CT	0.8	MRI				
10	− 5.8	CT	2.0	MRI				

Available imaging according to time-to-symptom-onset including first image available around symptom-onset, either CT or MRI. Patnr: patient number; timepoint: interval between imaging and onset of symptoms in years

In 2 cases 3 MRIs were available at − 6.1, − 2.8 and 0.2 and − 10.3, − 4.1 and 0.5 years to symptom onset respectively

## Evan’s index and width of 3rd ventricle

**Fig. 2:**
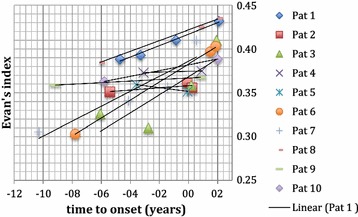
Change in Evan’s Index over time. Each patient is depicted separately. EI was above 0.3 in all patients and increased over time significantly. Time 0 is time of symptom onset

**Fig. 3:**
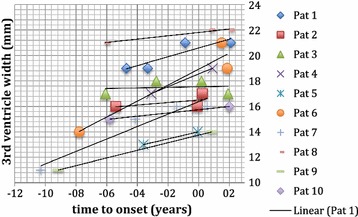
Change in third ventricle width over time. Each patient is depicted separately. Third ventricle width (in mm) was above 9 mm in all patients and increased over time. Time 0 is time of symptom onset

EI was ≥ 0.3 in all cases at all imaging times. Figure 2 shows EI over time. The values most prior to and closest to symptom onset were included. The same was done for 3VW (see Fig. 3). One patient suffered from bithalamic ischemia 4 years before symptom onset. This patient was not excluded because EI and 3VW had already been enlarged before the occurrence of the ischemic event. Six patients had available imaging data at time points before and after onset of symptoms. In the remaining four patients, the second examination was performed shortly before symptom onset. In these six patients, the mean value of EI was significantly higher after symptom onset than at the pre-symptomatic time point (mean value 0.39 ± 0.02 and 0.35 ± 0.03, respectively, p = 0.0354). 3VW did not show a significant difference between both time points (mean value 18.3 ± 3.0 and 15.8 ± 3.4, respectively).

## Tight high convexity

**Fig. 4:**
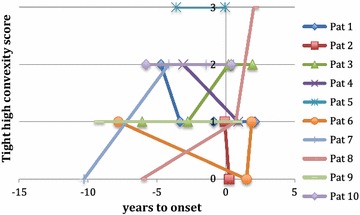
Change in tight high convexity over time. Each patient is depicted separately. THC varied through time within patients. Time 0 is time of symptom onset

Data on THC were available on the first image in nine patients. Seven of nine had a positive THC-score of 1, 2 or 3. The 2 patients who had no THC score of 0 in the first image at − 10 and − 6 years, did have mild score of 1 at − 4 years or a moderate score of 2, at − 1 year on the second available image. Two patients had mild THC of score 1 before symptom onset at − 5 and − 7 years, scored 0 after symptom onset at + 1.5 and 0 years. In the remaining 5 patients, THC score increased over time. All patients are depicted in Fig. 4.

## Other imaging findings

All patients with the relevant MRI (n = 7) showed periventricular and deep WMH. Only in 4 patients was MRI-data available at least 3 years before symptom-onset. The Fazekas score for both deep and periventricular WMH ranged from mild to severe (score 1–3) [42]. Aqueductal flow voids could be determined in 7 patients with T2-weighted sagittal MRI images and was present in all 7 cases. No major Sylvian fissure atrophy was seen.

## Discussion

This study shows that increased ventricular width, aqueductal flow void, deep and periventricular WMH and fluctuating THC precede symptom-onset of iNPH by at least 3 years. The presence or absence of THC seems to be a dynamic process varying in a non-linear fashion. This gives new insights into the pathogenesis of iNPH.

EI seems to be a robust measure for pre-symptomatic radiological changes in iNPH. EI increased significantly from the pre-symptomatic images to the time point of iNPH diagnosis. Extrapolation of the obtained data, assuming that EI change is linear over time, implies that the pathophysiological start of iNPH could be around 10–15 years before symptom onset. An on-going change in ventricular width might contradict the two-hit hypothesis on iNPH pathogenesis of the presence of benign external hydrocephalus in infancy followed by deep white matter ischemia later on as proposed by Bradley et al. [7, 43]. However, no proof of either theory exists to date. Further pre-symptomatic and long-term investigations are necessary. Moreover, it is conceivable that different subtypes of iNPH exist with different pathophysiological pathways.

Unlike Iseki et al. [5], our data show ventriculomegaly in almost all patients with only mild to moderate THC. A selection bias logically exists in the current study due to the retrospective nature and its inclusion criteria. The imaging studies at the pre-symptomatic time points were performed because of different neurological indications not connected to iNPH, whereas Iseki et al. have investigated a population-based cohort. Nevertheless, our data show a certain dynamic trend of THC in both increasing and decreasing throughout the years. These data suggest rather a variable than linear development of THC over time, possibly due to build up of internal CSF pressure followed by pressure decline after widening of the ventricles due to cell loss. Although the hypothesis of a transmantle gradient in pulsatile ICP in the lateral ventricles was disproven in 2010 [44], pressure was measured only in symptomatic patients. As CSF pulsatility index and CSF outflow resistance are increased in iNPH patients in the symptomatic phase [15, 16], increased ICP in the pre-symptomatic phase cannot be ruled out completely.

The presence of deep WMH together with the high prevalence of hypertension in our patients supports aspects of the hydrodynamic hypothesis of Greitz [45]. Approximately 50–60% of the 70–79 year old general population had hypertension according to a health survey 1988–1991 [46]. A more recent German survey showed that 60% of 50–59 year olds and 75% of 70–79 year olds suffer from hypertension [47]. Also signs of cerebrovascular disease were found in post-mortem and biopsy studies in iNPH patients [48–50]. Next to increasing blood pressure with age, there is a reduction of the dampening effect in cerebral arteries during aging due to stiffening of the arteries. This would further support the hydrodynamic theory [51]. In contrast, Qvarlander et al. did not find a significant difference in total cerebral artery inflow pulsatility index between healthy and iNPH patients [9]. It is thus unclear, if the extracranial arterial pulsatility reflects intracranial pulsatility, since the extracranial compartment is highly compliant as opposed to the intracranial compartment.

Transependymal edema or microangiopathic ischemia presenting as periventricular WMH was seen in all of our patients and existed in at least four patients before symptom onset. Previous data show that acetazolamide can reduce periventricular WMH with or without corresponding gait improvement in some but not all patients [19]. Acetazolamide inhibits CSF production in the choroid plexus, inhibits aquaporin-mediated water transport and normally leads to a CBF increase by vasodilation [52]. iNPH patients have an impaired cerebrovascular reactivity as shown before [53, 54]. The cerebrovascular reactivity was improved by acetazolamide in patients that showed clinical improvement after shunt surgery but not in the so-called non-responders [54]. All of our patients showed improved gait after shunt surgery or multiple lumbar punctures, while having had periventricular WMH for several years. Even years after symptom onset patients can improve after shunt surgery [55]. It remains unclear when the point of no return kicks in, where transependymal edema turns into ischemic tissue. To date no asymptomatic patient has received therapy for dilated lateral ventricles with typical iNPH imaging signs, as surgery has risks. Our data suggest that due to long-term progression a follow up of these asymptomatic patients with ventriculomegaly is necessary. These patients should be informed about the risk of developing iNPH symptoms in order to offer treatment at an early stage and preserve quality of life for as long as possible.

Limitations of this study include the retrospective nature of the investigation as well as the non-standardized documentation of clinical data, partially due to the fact that the patients were examined in two different departments. Furthermore, only low numbers of patients could be included due to the cut-off of available imaging at 3 years before symptom onset. The 3-year cutoff was set arbitrarily. Many patients already had symptoms 1–2 years before seeking medical advice. As symptoms start insidiously, imaging conducted at least 3 years before implied symptom-onset was considered to be realistically pre-symptomatic. The available imaging data was inconsistent, in some cases only CTs were available and not all parameters could be derived. This might have introduced biased assessments of the imaging data. However, length measurements as used in the EI and width of the third ventricle should be comparable between both modalities, and other measurements were based on rough scoring systems. Confounding is the fact that only iNPH-patients were investigated and not a population-dwelling cohort. However, it is likely that these changes are linked to iNPH, as patients in a previous study all developed iNPH-symptoms and very few asymptomatic participants were found after 10 years of scanning [5]. Due to the limited patient number, the statistical analysis can only give a preliminary overview. More longitudinal prospective research needs to be conducted similar to the work of Iseki et al. combining large cohort MRI data with clinical and neuropsychological data [5].

## Conclusions

In accordance with scarcely available literature radiological changes are present at least 3 years before onset of iNPH-symptoms. Evan’s index seems to be a robust measure for pre-symptomatic radiological changes. Extrapolating the data, the development of iNPH typical changes might be an insidious process and the development of tight high convexity might be a variable and non-linear process. Further studies with larger sample sizes are necessary to put these findings into the pathophysiological perspective of the development of iNPH.
